# Degradable Poly(ethylene oxide)-Like Plasma Polymer Films Used for the Controlled Release of Nisin

**DOI:** 10.3390/polym12061263

**Published:** 2020-06-01

**Authors:** Jaroslav Kousal, Jana Sedlaříková, Zuzana Kolářová-Rašková, Zdeněk Krtouš, Liliana Kučerová, Anna Hurajová, Mykhailo Vaidulych, Jan Hanuš, Marián Lehocký

**Affiliations:** 1 Faculty of Mathematics and Physics, Charles University, V Holešovičkách 2, 180 00 Prague, Czech Republic; jaroslav.kousal@mff.cuni.cz (J.K.); krtousz@gmail.com (Z.K.); vaydulym@yahoo.com (M.V.); jan.hanus@gmail.com (J.H.); 2 Faculty of Technology, Tomas Bata University in Zlín, Vavrečkova 275, 76001 Zlín, Czech Republic; lili.zl@seznam.cz (L.K.); lehocky@utb.cz (M.L.); 3 Centre of polymer systems, Tomas Bata University, Třída Tomáše Bati 5678, 76001 Zlín, Czech Republic; zraskova@cps.utb.cz (Z.K.-R.); hurajova@utb.cz (A.H.)

**Keywords:** poly (ethylene oxide), plasma polymerization, controlled permeation, nisin

## Abstract

Poly(ethylene oxide) (PEO)-like thin films were successfully prepared by plasma-assisted vapor thermal deposition (PAVTD). PEO powders with a molar weight (Mw) between 1500 g/mol and 600,000 g/mol were used as bulk precursors. The effect of Mw on the structural and surface properties was analyzed for PEO films prepared at a lower plasma power. Fourier transform (FTIR-ATR) spectroscopy showed that the molecular structure was well preserved regardless of the Mw of the precursors. The stronger impact of the process conditions (the presence/absence of plasma) was proved. Molecular weight polydispersity, as well as wettability, increased in the samples prepared at 5 W. The influence of deposition plasma power (0–30 W) on solubility and permeation properties was evaluated for a bulk precursor of Mw 1500 g/mol. The rate of thickness loss after immersion in water was found to be tunable in this way, with the films prepared at the highest plasma power showing higher stability. The effect of plasma power deposition conditions was also shown during the permeability study. Prepared PEO films were used as a cover, and permeation layers for biologically active nisin molecule and a controlled release of this bacteriocin into water was achieved.

## 1. Introduction

Polymer materials with antimicrobial properties are of the interests to many industrial and scientific sectors. For this purpose, a number of methods aiming at biomolecules’ attachment to polymer surfaces have been developed, such as adsorption, graft polymerization, or the formation of self-assembled monolayers [1,2,3,4]. Noncovalent interactions can occur at some types of interfaces; however, these are often not strong enough and other ways have to be applied to increase the coating stability [5]. The efficiency of the bioactive polymer system lies in the ability of the polymer material to selectively bind the active molecule and its consequent slow and controlled release [6,7].

Plasma technologies are clean, environmentally favourable methods that allow for the chemical and mechanical properties of polymers to be controlled. Plasma can be used for surface activation of a polymer substrate or by creating a thin, polymer-like layer on the surface [3,4,5,6,7,8,9,10,11]. So called “plasma polymerization” or plasma enhanced chemical vapor deposition (PECVD) uses plasma to activate and fragment an organic precursor in order to deposit a thin film. While such films (“plasma polymers”) only somewhat resemble classic polymers, they can be used, e.g., for improving the functionality of the surfaces for cell viability [8,12,13]. Plasma-polymerized poly(ethylene oxide) (PEO)-like films have been successfully demonstrated [11,14,15,16]. Polyester-based copolymers treated by two plasma methods, a diffuse coplanar surface barrier discharge (DCSBD) and capacitively coupled radio frequency discharge plasma under an argon atmosphere, were studied by Stloukal et al. [9] in order to eliminate the burst effect of the drug Temozolomide. The DCSBD technique was also used by Kolarova et al. [3] to modify the surface of a polyvinyl alcohol films crosslinked by glutaric acid and study its potential as a nisin carrier. The films prepared from polyethylene and polyethylene oxide (PE + PEO) enriched with nisin proved to be more effective against selected Gram-positive bacteria than systems without PEO [17]. Cui et al. investigated PEO-based fibers with encapsulated tea tree oil and plasma treatment and found that they exhibited increased antibacterial activity against *Escherichia coli* [18].

Plasma-assisted vapor thermal deposition (PAVTD) uses a polymer precursor, in powder form, that is thermally heated under a vacuum. Oligomeric fragments are released and can be re-polymerized in the plasma, producing a thin film. The advantage of this method is that the resulting plasma polymer is much more chemically similar to the classical polymer than films prepared by common vapor phase plasma polymerization. Moreover, the properties of the films are highly tunable by the plasma power which was demonstrated with polymers like polyimide [19], polyethylene [20], or polylactic acid [21]. Special interest has been paid to the poly(ethylene oxide) (PEO)-like films [22,23,24,25].

Based on some previous studies [17,18], poly(ethylene oxide) (PEO) was selected as the promising material known for its antifouling properties and the ability to protect surfaces from microbial attacks, which would be of great interest, especially for biomedical applications. The non-fouling properties of PEO-based surfaces are ascribed to the presence of ether units and flexible macromolecular chains in their structure. Moreover, PEO is able to decompose thermally, resulting in a mixture of linear oligomers that possess the similar chemical composition as the common PEO polymer [22].

In this context, the aim of the study was to evaluate the effect of PAVTD plasma polymerization method on the structural and surface properties of PEO thin films and to analyze their potential as carrier systems for active molecules. An amphiphilic low molecular weight polypeptide nisin was utilized since its high antimicrobial activity against a wide range of bacteria is proven.

## 2. Materials and Methods

### 2.1. Materials

Poly(ethylene oxide) (PEO) of various molecular weights (1500, 6000, 20,000, and 600,000 g/mol, referenced further as 1.5k, 6k, 20k, and 600k precursors), poly(vinyl alcohol) Mowiol^®^ 6-98, molar weight (Mw) ~47,000 g/mol (98–98.8 mol% hydrolysis), nisin standard from *Lactococcus lactis*, potassium chloride (KCl), ethylene glycol, and diiodomethane were obtained from Sigma-Aldrich (St. Louis, MO, USA). The bacterial species *Staphylococcus aureus* (CCM 4516) was provided by the Czech Collection of Microorganisms, and Mueller-Hinton agar was purchased from HiMedia Laboratories (Mumbai, India).

### 2.2. Thin Film Deposition

The film deposition was carried out following the process described in the study of Choukourov et al. [25], with some modifications used for the thin film deposition. The radio frequency (RF) powered (13.56 MHz, 0–30 W) electrode covered with a glass cover was placed 4 cm below a crucible containing a bulk powder precursor (source polymer) heated to 350 °C. Substrates (single-side polished silicon wafers, gold-coated silicon, aluminum foil, glass slides) were placed 10 cm above the crucible. The deposition was performed for 5 min in argon under a pressure of 4 Pa. The fragments of the bulk precursor were released from the crucible to the substrate either directly or through the plasma at a deposition rate of about 15 nm/min.

### 2.3. Characterization of Thin Films

A gel permeation chromatography (GPC) analysis of the films was performed on a HT-GPC 220 system (Agilent Technologies, Santa Clara, CA, USA). The separation and detection were carried out with PLgel MiniMix-E column (250 mm x 4.6 mm x 3 µm), the flow rate was 0.5 mL/min, the injection volume was 10 µL, and the mobile phase was a mixture of tetrahydrofuran and butylhydroxytoluene.

The FTIR-ATR spectra of the films were measured on Nicolet iS10 (Nicolet Instrument Corp., Waltham, MA, USA) with OMNIC software (Thermo Fisher Scientific, Waltham, MA, USA). Spectroscopic ellipsometry (M-2000 DI, J.A.Woollam, Lincoln, NE, USA) with a simple Cauchy optical dispersion model (CompleteEASE, J.A.Woollam) was used to measure the film thickness in dry (or dried) state. The XPS elemental composition of the films was measured using Phoibos 100 (Specs, Berlin, Germany).

The surface energy of the films was analyzed on the See System E (Advex Instruments, Brno, Czech Republic) using the static contact angle measurement method. A given amount of solvent (3 µL) was placed on the sample and the contact angle was measured. The surface energy was then evaluated with the help of the acid-base model. Water, ethylene glycol, and diiodomethane were used as reference solvents. The measurements were done in triplicate. The surface roughness was obtained using a scanning probe microscope SPM Ntegra Prima (NT-MDT, Moscow, Russia) in non-contact mode.

The zeta potential of PEO thin films was measured by a SurPASS Instruments electrokinetic analyzer (Anton Paar-SurPass, Graz, Austria). The adjustable gap cell was utilized with a 1 mM KCl electrolyte solution. The electrophoretic mobility and subsequent conversion to zeta potential were evaluated using the Smoluchowski model (Equation (1)):

(1) $$\zeta =\frac{\eta µ}{\varepsilon \varepsilon_{0}}\frac{\Delta V}{\Delta p},$$

where µ is the particle mobility, *η* is the viscosity, and *ε* is the permittivity, and a linear fit to the pressure–voltage curve indicates zeta potential.

### 2.4. Characterization of Permeation of Nisin

The nisin-containing layer (thickness ~45 nm) was formed via spin coating (1500 rpm) of the solution of polyvinyl alcohol (PVA) (5 wt %) with nisin (at a concentration of 125 µg/mL) on the glass substrates (2.5 × 2.5 cm). The samples were conditioned/incubated at room temperature for 20 min. Subsequently, a PEO-like PAVTD polymer layer (thickness ~70 nm) was deposited on these substrates (see Section 2.2).

To characterize nisin the release dynamics, the samples were placed in Petri dishes with 3 mL of 0.1% formic acid (for nisin stabilization) in a water solution, and aliquots were withdrawn at a defined time interval for LC-MS analysis. Nisin identification/quantification was carried out by high-performance liquid chromatography with quadrupole–time of flight mass detection (HPLC-QTOFMS). The column was Aeris Widepore XB-C8 (150 mm × 4.6 mm i.d., 3.6 µm, Phenomenex, CA, USA), and 0.1% formic acid and acetonitrile (Sigma-Aldrich) were used as a mobile phase. The flow rate was 1 mL/min and the injection volume was 5 µL. The nisin amount was quantified by means of a calibration curve. Nisin was analyzed by a gradient elution as a [M+5H]5+ (671.3163), [M+4H]4+ (838.8936).

The release kinetics of nisin before saturation was characterized by the modified Korsmeyer-Peppas model [26,27]:

(2) $$\frac{M_{t}}{M_{0}}=Kt^{n}+b,$$

where *M_t_* and *M*_0_ are the amounts of nisin in the sample at time t and 0, respectively; and *K*, *n*, and *b* are constants characterizing the release rate, kinetics exponent (*n* = 0.5 for Fickian diffusion through planar carrier) and initial burst/lag, respectively.

The retention of nisin bioactivity was monitored by inhibition zone tests. The samples of nisin-containing layer with PEO PAVTD film overcoat were immersed in 15 mL of demineralized water at laboratory temperature. Aliquots (1 mL) were withdrawn at defined intervals and microbiological analysis by an agar diffusion test using Staphylococcus aureus followed. One milliliter of bacterial suspension (10^6^ to 10^7^ cfu/mL) was transferred onto sterile Petri dishes containing a Mueller Hinton agar. An eluate (100 µL) was then inserted into the bored wells of 8 mm in diameter and incubated at 35 °C for 24 h. After the incubation period, the inhibition zone diameters around the samples were recorded. The antimicrobial susceptibility of nisin released through PEO films was compared with the antibacterial susceptibility of nisin solutions against *Staphylococcus aureus* where the threshold concentration of nisin to induce visible inhibition zone was 2 ug/ml.

## 3. Results

### 3.1. Influence of Molar Weight of Precursor Material on Properties of Thin Films

The dependence of the PAVTD thin film properties on the Mw of the bulk precursor was studied for materials deposited solely by thermal degradation/evaporation without plasma (0 W) or with mild plasma repolymerization (RF power 5 W).

The results of GPC analysis are shown in Table 1. It is known that in the GPC method the separation of macromolecules according to their hydrodynamic volume occurs. Whereas smaller molecules penetrate into the pores resulting in longer elution time, large molecules pass through the column quickly. In a polymer structure, branched macromolecules of lower Mw can be evaluated as larger linear molecules due to the production of an equivalent signal [24]. Regarding this fact, a wider Mw distribution can be expected in the plasma deposited PEO films. As shown in Table 1, the films formed without plasma have a lower Mw than plasma polymers formed at 5 W. The polydispersity of films prepared without plasma increased in comparison with precursors, where PDI ranged from 1.1 to 1.4. Similar to Mw, the polydispersity was even higher in the samples prepared at 5 W. There was no clear dependence of the Mw of the films on the Mw of the precursors. The Mw of these films was several thousand g/mol in all cases. There was some increase in the Mw of the plasma (re)polymerized films with the increasing Mw of the precursors; however, the high polydispersity reveals the presence of both low Mw and very high Mw fragments in the plasma polymer layers.

**Figure 1:**
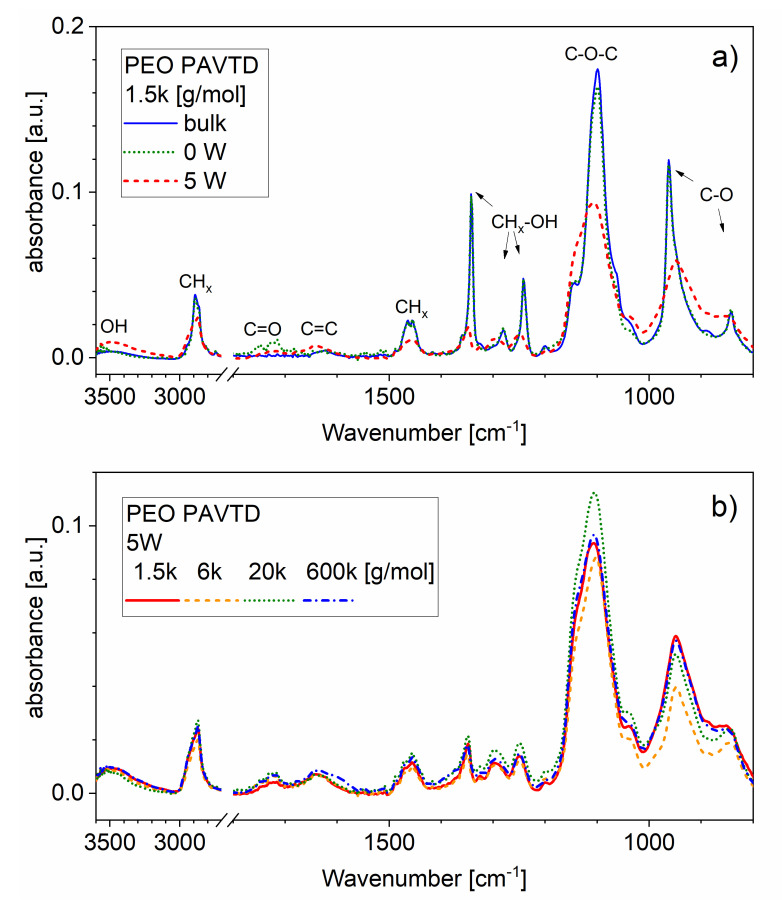
Infrared spectra of the films. (**a**) Comparison of the original polymer precursor (1500 g/mol) with the deposited films; (**b**) comparison of poly(ethylene oxide) (PEO)-like plasma polymer (RF power 5 W) films prepared from polymer precursors with varying molar weight (Mw).

The infrared spectra of the films (Figure 1a) show the peaks typical for bulk PEO as found in the literature [24]. The spectra are dominated by C–O and CH_x_ bonds with a minimal amount of C=O bonds. Plasma polymerized films show an increased amount of –OH, C=O, and C=C bonds and decreased amount of C–O–C bonds. Generally, the PEO-like structure is retained in the films, with visible broadening of the peaks compared to the bulk precursor, which could correspond to the change from crystalline to amorphous phase. This is more pronounced in plasma-polymerized films, corresponding to a higher fragmentation of the polymer chains suggested by GPC analysis. This trend is independent of the Mw of the bulk precursor (Figure 1b).

The XPS analysis of the films gave results that are consistent with the PEO-like structure. In all cases, the C/O ratio was found to be 2.0 ± 0.1, as in bulk PEO. However, the C1s peak decomposition revealed about 5% of C=O bonds (only the film prepared from the 6k precursor had a proportion this close to 10%) and the decrease of the C–O–C bonds proportionate with power, with a simultaneous increase in the number of C–C/C–H bonds, as indicated by the FTIR analysis. In the films prepared without plasma, the C–O–(C) group contributed to about 80% of the carbon peak (with a slight decrease with the increasing Mw of the precursor, from 85% for 1.5k to 78% for 600k precursor). The number of C–O–(C) bonds in the films decreased with increasing plasma power (75% at 5 W, 70% at 30 W). The O1s spectra confirmed the C=O/C–O bond ratio found from the analysis of the C1s peak. The film composition is similar to that in previous studies [23,24] and only shifted in plasma power levels due to the differences in the deposition setup.

**Figure 2:**
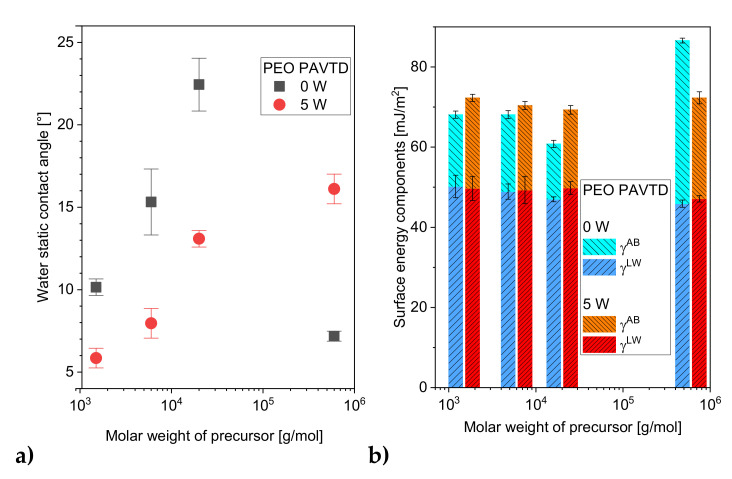
Surface properties of the films prepared from set of bulk precursors with varying Mw prepared with and without plasma power. (**a**) Water contact angle; (**b**) surface energy components.

The wettability and surface energy of prepared PEO-like plasma polymer films are shown in Figure 2. As expected, the higher surface energy is correlated with higher wettability [28]. The water contact angles of the films (Figure 2a) increased with the Mw of bulk precursors and decreased for plasma polymerized films, except for the film prepared from the precursor with the highest Mw. The reason could lie in the fact that higher Mw led to lower surface roughness, which caused enhanced wettability [29]. The surface roughness slightly increases with the Mw of the precursor, from 3 nm RMS roughness for the 1.5k precursor to 10 nm for the 600k precursor. The roughness of the films prepared under 5 W plasma power somewhat lower (see Table 2). A similar trend has been observed for films prepared using PAVTD of polyimide [19]. The fact that the most significant drop in relative surface roughness was found in the samples prepared from 600k at 0 and 5 W helps to explain the observed decrease in contact angle.

Surface energy (SFE) values were calculated using an acid–base model with water, diiodomethane, and ethylene glycol as reference liquids. The total surface energy *γ^total^* is split into dispersion, *γ^LW^*, and a polar part, *γ^AB^* (Equation (3)).

(3) $$\gamma^{total}=\gamma^{LW}+\gamma^{AB}.$$

As seen in Figure 2b, no significant effect of Mw was observed. SFE values were around 60 to 70 mJ/m^2^, regardless of the molecular weight of the polymer. Only in the case of the highest Mw was a slight increase in surface energy observed. When films prepared without plasma (0 W) and with 5 W RF power were compared, an increase in SFE and in the *γ^AB^* component was found for samples prepared from precursors 1.5k, 6k, and 20k. The film prepared from the 600k precursor exhibited a drop in the SFE and *γ^AB^* values, from 87 to 72 mJ/m^2^ and 41 to 25 mJ/m^2^, respectively. The contact angle and surface energy measurement showed a bigger effect of the plasma power than of the Mw of the precursor. A similar trend was observed in the study [3] where polyvinyl alcohol films treated with surface plasma dielectric coplanar surface barrier discharge were investigated.

Solid surfaces that are in contact with a solution are surrounded by a so-called electrical double layer that is composed of compact and diffuse parts. The zeta (ζ) potential is the electrostatic potential existing at the boundary dividing these two layers. Measurement of ζ potential is one of the important electrokinetic characterization methods that provide information on the surface hydrophilicity, stability, and thus the preconditions for further potential interactions with active agents, surfactants, vitamins, etc. [3,30].

**Figure 3:**
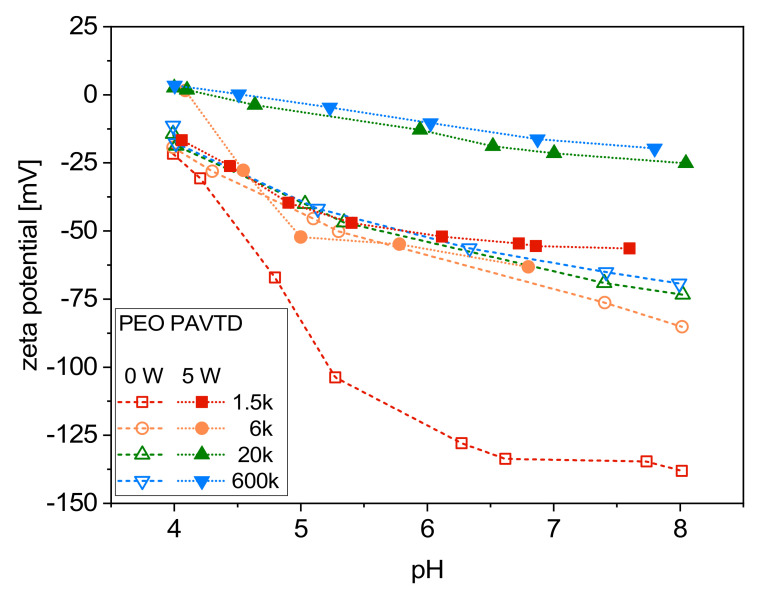
Zeta potential curves for the films prepared from set of bulk precursors with varying Mw prepared with and without plasma power.

The effect of precursor Mw and plasma power during deposition in the pH range from 4 to 8 is shown in Figure 3. Generally, almost all the samples showed negative zeta potential values depending on the Mw and RF power applied during the preparation. This can lead to the assumption that the film surface was widely covered with ionic or charged groups [31]. The resultant plasma film structure can be more prone to water absorption, which could lead to enhanced dissociation and the development of a negative charge [32]. Higher zeta potential values (+3.4 to −25 mV) were recorded for the samples prepared from PEO of Mw 20k and 600k at 5 W. The zeta potential of the films from lower Mw, 1.5k and 6k, prepared under the same conditions were between +1.65 and −63 mV—very close to the values of the samples prepared without plasma (0 W). A relatively significant difference is visible in the sample prepared from the precursor with the lowest Mw (1500 g/mol) without plasma power, where a steep decrease of the zeta potential to almost −140 mV was recorded. In line with the XPS results, no unambiguous statement can be made about the presence of COO– groups. Thus, the result could be because this layer was mostly washed off during the measurement when comparatively high pressure was applied.

Obviously, plasma polymers showed different surface properties to films formed without plasma (at a power of 0 W), which could be utilized for the attachment of various active components. Following some previous studies [23,25], where PEO with Mw 1.5k has been investigated, this was chosen as a precursor for the films that were used for further degradability and nisin release experiments.

### 3.2. Control of Film Degradability and Nisin Release

The effect of the plasma polymerization process on the film degradability and conditions of nisin bioactive molecule release was studied. Typically, a bioactive compound release can proceed by diffusion through a biodegradable polymer matrix and/or due to the degradation of the polymer backbone, followed by a matrix erosion. In the case of a classical release of encapsulated substances, a high concentration gradient between the active molecule and aqueous media is predominant. This phenomenon leads to a significant rise in concentration during the initial phase (the so-called burst effect), which is highly undesirable in applications where a continuous longer-term controlled release is required [33]. This can be achieved by special surface treatment, such as plasma deposition.

This part of the study was performed using the PEO polymer precursor with a Mw of 1500 g/mol since it was the most promising one for the regulation of PEO-like layer properties by deposition conditions (Section 3.1).

**Figure 4:**
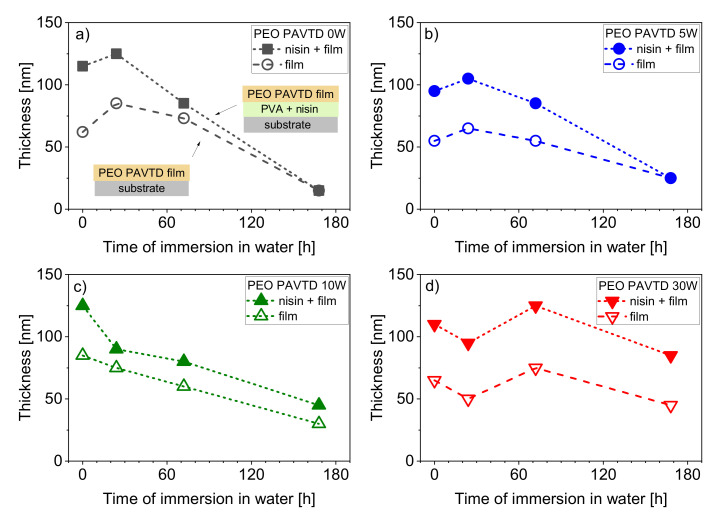
Total thickness of the PEO plasma-assisted vapor thermal deposition (PAVTD) films and of the systems of nisin-containing layer with PEO PAVTD films overcoat during immersion in water. PEO PAVTD films were prepared using precursor with Mw 1500 g/mol at (**a**) 0 W, (**b**) 5 W, (**c**) 10 W, and (**d**) 30 W.

The samples with and without a nisin-containing layer were coated simultaneously with PEO-like films. The samples from batches with plasma power during the deposition of 0–30 W were then immersed in water, and their dry thickness was monitored as deposited and after 24, 72, and 168 h of immersion (Figure 4).

An initial increase in thickness of PEO-like films was observed in the films prepared at a lower plasma power (Figure 4a,b), probably due to the swelling of the PEO-like films. After a longer immersion in water, the films started to lose thickness, possibly because of the washing out the shorter polymer fragments (oligomers). This thickness loss is more pronounced for films prepared without plasma or with a low (5 W) plasma power. The films prepared at 30 W appear to be the most stable. The difference in thickness between films with and without a nisin-containing layer is a marker of the dissolving of the PVA, along with the expected nisin release. For samples with a PEO-like overcoat prepared at low powers, this difference diminishes faster during the immersion in water, suggesting a faster nisin release.

**Figure 5:**
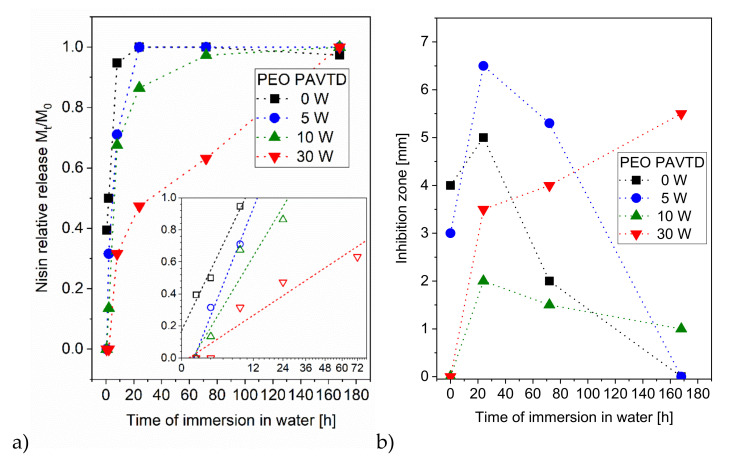
Characterization of the release of nisin from the nisin-containing layers with PEO PAVTD films overcoat during immersion in water. (**a**) Relative release of nisin into water (the power law fits of the data are shown as dashed lines in the inset with square-root scale x axis); (**b**) antimicrobial activity of the films characterized by the width of the inhibition zone. The inhibition zones were evaluated as the average from three measurements. Since the standard deviation was lower than 10%, the error bars were not included in the microbiological analyses.

A nisin profile release from PEO films was monitored over 168 h at 37 °C, and concentrations were analyzed by LC-MS. As seen in Figure 5a, the nisin release from all tested samples showed a nonlinear time profile, with basically all nisin being released within a week, so the trapping of nisin inside the PVA carrier layer [34] can be excluded. From samples prepared at a low plasma power (0–10 W), most of the nisin was released within the first 8 h. However, even plasma treatment at 0 W led to a significant slowdown of nisin release compared to the samples without PEO cover (where most of the nisin was released within minutes).

The acquired experimental data were analyzed by applying the power law equation (Equation (2)). The equation is commonly considered to be valid for *M_t_/M*_0_ < 0.6 [27], but, due to the limited number of data points given by the time constraints of the measurements, we included the data points up to *M_t_/M*_0_ < 0.95. For simplicity, we fixed the kinetics exponent at *n* = 0.5 as for classical diffusion. Therefore the fitted parameters (Table 3) are only indicative. The rate constant K drops with plasma power during film preparation from 0.3 h^−1/2^ (0 W) to 0.09 h^−1/2^ (30 W). The sign of burst/lag constant b indicates that nisin release through the film prepared without plasma undergoes an initial burst, while the release through the plasma (re)polymerized films shows a rather small lag.

Microbiological evaluation by the agar diffusion (inhibition zone) method confirmed the continued biological activity of nisin after the release from the samples. The time dynamics of antibacterial effects were dependent on the plasma power during PEO-like coating deposition (Figure 5b). The samples prepared at 0 W and 5 W showed decreasing antibacterial activity after the first 24 h, corresponding to the initial burst effect with the subsequent lack of free bioactive nisin. The samples with coatings prepared at a higher RF power (10 W and 30 W) exhibited a steady or increasing trend in suppressing the bacterial growth, mitigating the burst effect. These findings correspond to the above-mentioned results obtained by LC-MS.

## 4. Conclusions

PEO-like thin films were prepared by plasma-assisted vapor thermal deposition from PEO powder precursors of Mw from 1500 to 600,000 g/mol. Only a slight impact of precursor Mw on the surface properties and chemical structure of the films was observed. The PEO precursor with the lowest Mw was selected for investigating the plasma power effect (0–30 W) on the degradation and release of nisin. Results showed that the films prepared by using higher plasma power are more suitable for controlled release of nisin than films prepared at a lower power. At the same time, the nisin release kinetics from PEO-like film was shown to be tunable by the deposition conditions, which can be utilized for the elimination of the undesirable burst effect.

## Figures and Tables

**Table 1:** Molar weight (Mw) of the films prepared from various bulk precursors with and without plasma.

	Molar Weight (Mw, g/mol)		Polydispersity (PDI)		
Bulk Polymer	PAVTD Film				
	0 W	5 W	bulk	0 W	5 W
1.5k	5500	6500	1.26	1.5	2.6
6k	6000	15000	1.56	1.7	3.2
20k	12000	60000	1.31	2.4	4.6
600k	2300	60000	1.07	1.8	3.8

**Table 2:** Roughness of the films prepared from various bulk precursors with and without plasma.

PEO PAVTD	AFM RMS Roughness [nm]	
Precursor	0 W	5 W
1.5k	2.9 ± 0.2	1.5 ± 0.1
6k	6.7 ± 3.8	6.1 ± 1.9
20k	8.7 ± 1.3	8.7 ± 1.6
600k	9.8 ± 0.4	3.6 ± 1.0

**Table 3:** Fitted values of parameters of the model equation of nisin release data.

PEO PAVTD	K [h^−1/2^]	b	R^2^
0 W	0.27 ± 0.07	0.17 ± 0.13	0.977
5 W	0.33 ± 0.07	−0.20 ± 0.13	0.984
10 W	0.22 ± 0.03	−0.11 ± 0.10	0.918
30 W	0.09 ± 0.02	−0.03 ± 0.07	0.902
